# Tertiary and quaternary structural basis of oxygen affinity in human hemoglobin as revealed by multiscale simulations

**DOI:** 10.1038/s41598-017-11259-0

**Published:** 2017-09-07

**Authors:** Mauro Bringas, Ariel A. Petruk, Darío A. Estrin, Luciana Capece, Marcelo A. Martí

**Affiliations:** 1 Departamento de Química Inorgánica, Analítica y Química Física, Facultad de Ciencias Exactas y Naturales, Universidad de Buenos Aires, Ciudad de Buenos Aires, C1428EHA Argentina; 2 Instituto de Química Física de los Materiales, Medio Ambiente y Energía, CONICET, Ciudad de Buenos Aires, C1428EHA Argentina; 3 Departamento de Química Biológica, Facultad de Ciencias Exactas y Naturales, Universidad de Buenos Aires, Ciudad de Buenos Aires, C1428EHA Argentina; 4 Instituto de Química Biológica de la Facultad de Ciencias Exactas y Naturales, CONICET, Ciudad de Buenos Aires, C1428EHA Argentina

## Abstract

Human hemoglobin (Hb) is a benchmark protein of structural biology that shaped our view of allosterism over 60 years ago, with the introduction of the MWC model based on Perutz structures of the oxy(R) and deoxy(T) states and the more recent Tertiary Two-State model that proposed the existence of individual subunit states -“r” and “t”-, whose structure is yet unknown. Cooperative oxygen binding is essential for Hb function, and despite decades of research there are still open questions related to how tertiary and quaternary changes regulate oxygen affinity. In the present work, we have determined the free energy profiles of oxygen migration and for HisE7 gate opening, with QM/MM calculations of the oxygen binding energy in order to address the influence of tertiary differences in the control of oxygen affinity. Our results show that in the α subunit the low to high affinity transition is achieved by a proximal effect that mostly affects oxygen dissociation and is the driving force of the allosteric transition, while in the β subunit the affinity change results from a complex interplay of proximal and distal effects, including an increase in the HE7 gate opening, that as shown by free energy profiles promotes oxygen uptake.

## Introduction

**Figure 1:**
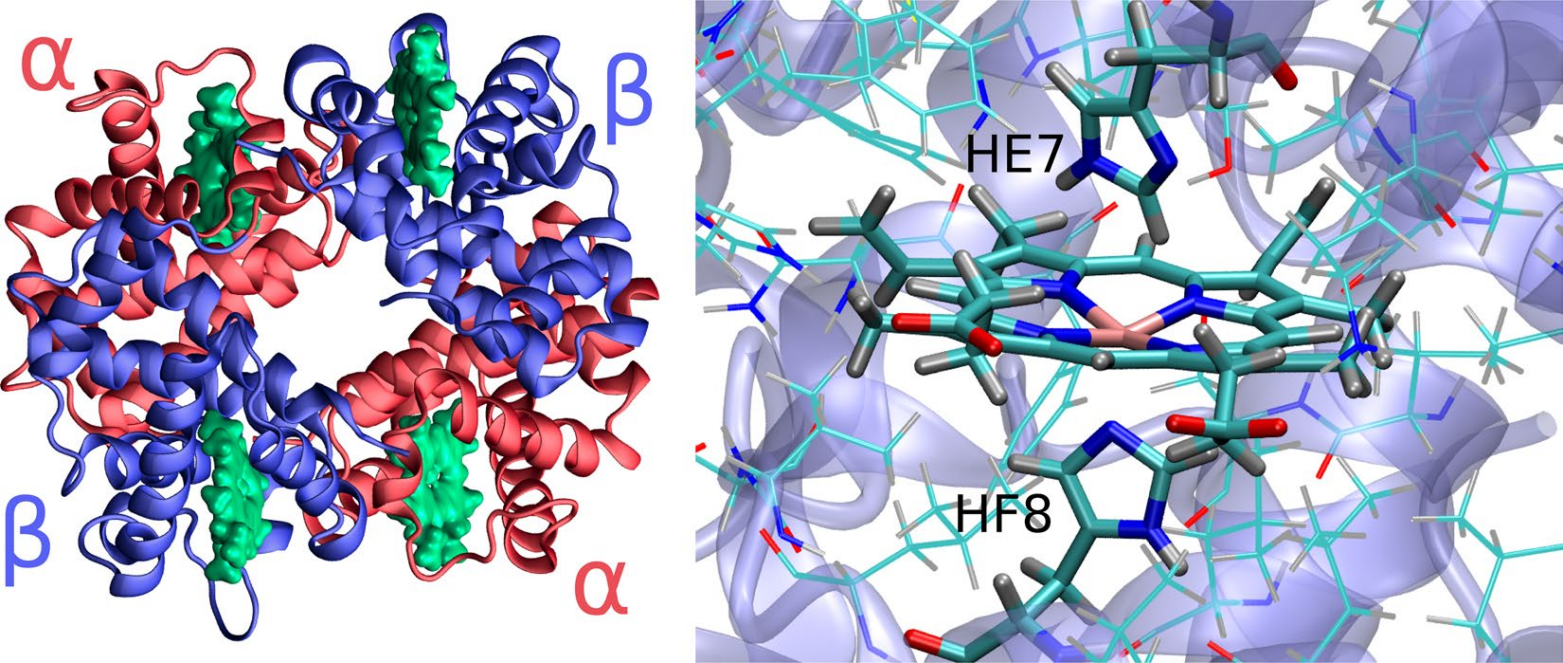
Hemoglobin tetrameric alpha helical structure on the left. Schematic representation of Hb heme environment, highlighting both proximal and distal histidines, on the right.

Hemoglobin (Hb) and the simpler related protein, myoglobin (Mb), are among the most thoroughly studied heme proteins, being benchmarks cases for many experimental and theoretical developments in a wide range of fields covering structural biology, bioinorganic chemistry and genetics, among others. Hb is a tetrameric protein composed of four globular (Mb-like) subunits, two of each kind, named α and β. Each subunit is composed of 8 α-helices, named A to H, connected by short non helical regions and holds the prosthetic heme group (see Fig. 1) coordinated to the protein through histidine F8 (HF8), called the proximal histidine. The other key residue related to its function is the histidine present in position E7, the distal histidine (HE7)^1, 2^. Hb function is tightly related to heme iron reactivity towards small molecules, specially oxygen, and even though its history spans almost a century, several questions remain open^3, 4^. Since Hb is a tetrameric protein, the key to its physiological role concerns the cooperatively regulated differential oxygen affinity between the T(tense, low affinity) and R (relaxed, high affinity states), and how it relates to both quaternary and tertiary structural changes.

Allosteric and structural based models used to understand oxygen affinity in Hb were born with the initial Hb crystal structures obtained by Perutz for both oxy(R) and deoxy(T) Hb states^5, 6^ and later developed into general allosteric MWC model by Monod *et al*.^7^. This symmetry-based model relies on the existence of an equilibrium between the two quaternary R and T states, and assumes equivalence of all four subunits. The fact that Hb contains two chemically different subunits α and β and presents a single two-fold axis of symmetry, lead to extensions of the MWC model, as well as alternative formulations such as the sequential model, introduced by Pauling^8^ later elaborated by Nemerthy *et al*. and usually known as NFK model^9^. Perutz also spent several years looking for structural reasons underlying the different states oxygen affinity and proposing that oxygen binding to the T state results in an in-plane movement of the heme iron, with the concomitant motion of the proximal HisF8 and the associated F-helix, resulting in the breaking of several key intersubunit salt bridges that would promote the transition to the R-state, leading to the so called stereochemical model^5, 6, 10, 11^. Decades later, Szabo and Karplus revisited this model and included statistical thermodynamic and energetic considerations into the stereochemical model, proposing that low oxygen affinity of the subunits in the T state, arose from the strain at the ‘allosteric core’, mainly the steric repulsion between HisF8 and the porphyrin^12, 13^. All these models focused on quaternary transitions and paid little attention to differences between type of subunits and tertiary transitions.

The latest -more recent- model, which explicitly includes tertiary differences, is known as the Tertiary Two State (TTS) model, as originally proposed by Henry *et al*.^14^ and was motivated by observations of the oxygen affinity in quaternary trapped states^15, 16^ and spectroscopic evidence of pure tertiary transitions^17^. The TTS model postulates that high and low affinity conformations of individual subunits, which are called “r” and “t”, exist in equilibrium within each quaternary structure, and allows -in opposition to MWC- incomplete coupling between tertiary and quaternary transitions. The TTS model recently received strong support from CO rebinding kinetic experiments in gel encapsulated Hb by Viappiani *et al*.^18^.

At this point it is important to note that although the described Hb models allow for the interpretation and assignment of oxygen binding data, and relate them with some structural features, a clear microscopic picture of how conformation of each tertiary/quaternary state control oxygen differential affinity is still missing. In this context, several basic questions like “what is the structural origin of the difference in affinity for oxygen binding to t and r?” or, “what are the relative contributions of the proximal strain and intersubunit contacts?” remain unanswered.

Chemically speaking, oxygen -and any other ligand- affinity in heme proteins, can be rationalized in terms of two processes: oxygen association, characterized by the bimolecular rate constant termed “k_on_”, and oxygen release, which is characterized by the dissociation rate constant “k_off_”^19^. Kinetic measurements using hybrid Hbs, in which alternatively α or β hemes are replaced by porphyrin containing metals that do not bind O_2_ or CO (Cr^II^, Mn^II^, Ni^II^ and Mg^II^), allowed estimation of each subunit quaternary dependent rate constants, yielding values of k_on_(α) = 11μM^−1^s^−1^, k_on_(β) = 5μMs^−1^, k_off_(α) = 3700 s^−1^ and k_off_(β) = 1800s^−1^ of the T state, and k_on_(α) = 36μM^−1^s^−1^, k_on_(β) = 76μM^−1^s^−1^, k_off_(α) = 16 s^−1^ and k_off_(β) = 32 s^−1^ for the R state^20^. In globins, ligand association is controlled by migration of the ligand from the bulk solvent into the active site through the protein gates and/or tunnels, a process -that as shown in our previous works, can be characterized by computing the corresponding free energy profile (FEP)^21–23^. In particular, for Mb, we showed that the depth of the free energy well in the distal pocket, which is directly related to k_on_, is controlled by the HE7 “gate” conformation^24^. Dissociation, on the other hand, is controlled by disruption of the protein bound ligand interactions, which, as studied in previous works^25–27^, can be accurately determined using hybrid quantum mechanics/molecular mechanics (QM/MM) based methods and directly correlated with observed k_off_ values^25^. More important, both calculations, allow a deep understanding of the structural reasons that control each process and thus ultimately determine globin ligand affinity^28^.

In the present work we have combined the above mentioned type of classical molecular dynamics (MD) and multiscale QM/MM simulations to address the influence of tertiary structures in the control oxygen affinity, and the molecular basis of the difference between each subunit type (α vs β) and quaternary state (R vs T) oxygen affinity. Our results, discussed in the context of Hb allosteric models, show how proximal effects that govern α chain differential affinity are the driving force of the allosteric transition, and how proximal and distal effects are combined to modulate β chain affinity.

## Computational methods

### Starting Structures

Two representative high resolution X-ray crystallographic structures were taken as starting points for all simulations, corresponding to the deoxy (T) and oxy (R) forms of Hb, (PDB codes 2dn1 and 2dn2 respectively)^29^. These, as well as other lower resolution crystal structures corresponding to the same quaternary states, have been successfully used in previous computational studies to represent each quaternary structure^2, 3, 30–32^. Standard protonation states at physiological pH were assigned to all ionizable residues (Asp, Glu, Lys and Arg) paying special attention to the protonation of histidines, which were assigned on the basis of the hydrogen-bond pattern with neighboring residues. In particular, β146 histidine is known to be a key factor in the quaternary T state stabilization, was simulated as the protonated (HIP) tautomer^30^. For the proximal heme-bound histidine (HF8), protonation was chosen to be in the Nδ position, since this is the protonation state that allows coordination to the iron. For distal histidine, (HE7) all three protonation states (HID, HIP and HIE) were simulated and analyzed (see below).

To build the proximal/distal single site mutants, we used the same protocol as in Capece *et al*.^26^. Briefly, HE7G was built by simply removing the HE7 side chain and adding a hydrogen atom in place of the Cα-Cβ bond. HF8Gly + Im mutant was built removing the His Cβ methylene and completing the resulting Gly and Imidazol with the corresponding hydrogen atoms. Both mutants were built starting from the corresponding oxygenated classically optimized structures for each subunit/state.

### Classical Molecular Dynamics Simulations

All Molecular Dynamics simulations were performed using the PMEMD module of the Amber14 package. The Amber99SB force field^33^ was used for all residues but the heme, whose parameters were developed and thoroughly tested by our group in previous works^27, 34–36^ and are available in the SI. It is important to note that our developed heme parameters are coordination and oxidation state specific and are specially designed to reproduce the experimentally observed different heme conformations and interactions with the protein matrix, allowing the performance of stable MD simulations of both R and T states of Hb (see below)^28, 37^. Complete starting structures were finally immersed in an octahedral box of TIP3P waters^38^. All simulations were performed using periodic boundary conditions and Ewald sums to treat long range electrostatic interactions^39^, the SHAKE algorithm to keep bonds involving hydrogen atoms at their equilibrium length^40^, a 2 fs time step for the integration of Newton’s equations, and the Berendsen thermostat and barostat to control the system temperature and pressure respectively^41^. Equilibration consisted of an energy minimization of the initial structures, followed by a slow, 2 ns long heating up to 300 K (in the NVT ensemble), and a final 300 ps long density equilibration procedure (in the NPT ensemble). Production MD runs consisted of 100 ns long trajectories. Frames were collected at 20 ps intervals, which were subsequently used to analyze the production trajectories.

### QM–MM calculations

The initial structures for the QM–MM calculations corresponded to classically optimized starting structures described above, thus they remain close to the crystallographic conformation. Full hybrid QM–MM geometry optimizations were performed using a conjugate gradient algorithm. Only residues located less than 10 Å apart from the heme reactive center were allowed to move freely in the QM–MM runs. All QM–MM computations were performed at the DFT level using the generalized gradient approximation to the exchange-correlation energy proposed by Perdew *et al*.^42^ with the SIESTA code^43^ which has been extensively used by our group to study heme proteins. Basis functions consist of localized (numerical) pseudo-atomic orbitals, projected on a real space grid to compute the Hartree potential and exchange correlation potential matrix elements. For all atoms basis sets of double zeta plus polarization quality were employed with a pseudoatomic orbital energy shift of 25 meV and a grid cutoff of 150 Ry. Further details can be found elsewhere^44–46^. In all cases, the iron porphyrinate, the O_2_ ligand and the imidazole group of the proximal histidine of one Hb subunit were selected as the quantum subsystem. The rest of the protein, together with water molecules, were treated classically.

To analyze structural effects on oxygen dissociation, we computed for each case the O_2_ binding energy (ΔEO_2_), which is defined as:

1 $${\rm{\Delta }}{E}_{O2}={E}_{Heme-O2}-({E}_{O2}+{E}_{Heme})$$

where E_Heme–O2_ is the energy of the oxy-form of the protein, E_Heme_ is the energy of the deoxy-form, and E_O2_ is the energy of the isolated oxygen molecule. This strategy was successfully used in our previous studies, particularly for determining distal and proximal effects on the oxygen affinity in globins^26^. Moreover, recent works from our group showed that QMMM calculated ΔE_O2_ can be used to estimate k_off_ based on an statistical model, where the ΔE_O2_ and k_off_ are expressed relatively to those of a free heme.^47^. The corresponding results show that the accuracy of the predicted dissociation rate values is around one order of magnitude and, most important, to correctly assign the order of the predictive experimental constants.^25, 27^ This correlation was already applied to the study of many globins oxygen affinity^25^. In other words, larger ΔE_O2_ will definitely result in smaller koff values and vice-versa.

### Free Energy profiles

In order to evaluate the oxygen association process, we calculated first the free energy profiles (FEP) for O_2_ migration from the solvent to the distal pocket using multiple steered molecular dynamics (MSMD), as performed by Boechi *et al*. for Mb^24^. Briefly, in MSMD an external force, described as an harmonic potential, is added to the system Hamiltonian whose equilibrium value moves at an arbitrary velocity and thus steers the system towards the desired state. Integrating the external force allows determination of the irreversible work profile W(i) along the selected reaction coordinate. Finally, FEP is obtained by exponential averaging multiple work profiles, starting from different initial equilibrated microconfigurations, employing Jarzynski’s equation.^48, 49^

2 $${\langle {e}^{-{W}_{i}/{k}_{B}.T}\rangle }_{i}={e}^{-{\rm{\Delta }}G/{k}_{B}.T}$$

where the brackets indicate an infinite average over the collected works (*W*_*i*_), *k*_*B*_ the Boltzmann constant and T the temperature, and *ΔG* the free energy change. Error estimation was performed according to Bustamante *et al*.^50^, considering the FEP statistical uncertainty as the square root of the Mean Square Error (MSE), computed according to $MSE={\sigma }^{2}(N)+{B}^{2}(N)$, where *σ* ^2^ corresponds to the variance of the calculated work profiles, *B* ^2^ to the Bias of the Jarzynski’s estimator, and *N* the number of MSMD trajectories. (see reference^50^ for details). For each profile, we performed 40 trajectories using as reaction coordinate the Fe-O distance and a 150 kcalmol^−1^Å^−2^ force constant. The pulling speed was chosen to be 0.25 Åns^−1^ as optimized for these type of calculations in previous works^23^. Due to the large extension of the reaction coordinate, simulations were divided in two regions: the first region ranges from the solvent to the first minimum, which corresponds to a secondary docking site in the migration pathway. The second region covers the reaction coordinate from the docking site until the oxygen reaches the sixth coordination position. This strategy allows to keep the ligand close to the migration pathway, avoiding unproductive trajectories. In the four cases, the resulting profiles were combined in order to overlap as best as possible the free energy minimum (corresponding to the docking site) and the slope corresponding to the barrier from the docking site to the iron bound state. To qualitatively describe the Fe-ligand bond formation when the ligands gets too close to the heme we added a Morse potential for the Fe-O bond. We used 1.8 Å as the equilibrium distance, a width control parameter of 5.6 Å^−1^ and a well depth of 10 kcalmol^−1^. It is also important to note, that although charge distribution of the heme and its ligand are altered during the binding process, and ligand parameters have been proposed and implemented^51, 52^ to account for this fact, our purpose is to evaluate the migration free energy profile at distances which are significantly larger than Fe-O bond where these charge redistribution effects are not expected to be of relevance, as previously shown. Thus we used unbound heme and oxygen parameters during the whole SMD process. Finally, in order to avoid unproductive trajectories due to protein rotation and free movement of the ligand in water, all the SMD simulations were started with the oxygen molecule completely solvated but next to the protein surface (at the entrance of the tunnel).

Secondly, and in order to complete the free energy landscape for O_2_ uptake, we also computed the FEP associated to the opening of the HE7 gate using umbrella sampling simulations, following a similar protocol as in our previous work for Mb^53^. The chosen reaction coordinate was the dihedral angle C-Cα-Cβ-Cγ of HE7, and was sampled from 60 degrees (closed conformation) to 160 degrees approximately (open conformation). The reaction coordinate was explored in 10 consecutive windows.

## Results

The results are organized as follows: in the first section we present QM/MM analysis of oxygenated structures and their O_2_ binding energy for wild type as well as proximal and distal histidine mutants. Secondly, we show the results obtained in equilibrium molecular dynamics simulations on the R and T states for the tetrameric structures. Lastly, we analyze the process of O_2_ entrance to the distal cavity by means of free energy calculations.

### QM/MM oxygen binding energy in α and β subunits of R and T hemoglobin

#### Wild type (wt) Crystal and solution structures

To start unraveling the tertiary structural determinants of the differential oxygen affinity in α and β subunits of R and T Hb (from now on called αR, αT βR and βT), we computed the optimized oxygenated structure, and the oxygen binding energy (ΔE_O2_) for each monomer in the following crystal structures 2dn1(R) and 2dn2(T), which are commonly used as R and T quaternary references structures. ΔE_O2_ is related with the experimental observed k_off_ value, and is able to accurately describe and dissect the energy required to break the bound oxygen-protein (both Fe-O_2_ and intermolecular protein O_2_ interactions). Thus, proteins (or subunits) with large negative ΔE_O2_ display small values of k_off_, while those displaying smaller ΔE_O2_ values exhibit large k_off_ values, as previously shown by our group for a large set of globins^27^. The results are presented in Table 1.

**Table 1:** Energetic and geometric characterization of α and β HbA subunits from both R and T crystallographic structures.

Parameter	Monomer and Quaternary State			
	αT	αR	βT	βR
ΔEo_2_/kcal mol^−1^	−23.1	−30.2	−21.7	−30.7
dFe-O/Å	1.77	1.77	1.84	1.83
dO-O/Å	1.30	1.30	1.30	1.30
dFe-NHis Oxy/Å	2.19	2.12	2.08	2.09
dFe-NHis/Å	2.20	2.12	2.09	2.12
qO_2_/e-	−0.285	−0.270	−0.326	−0.308
HF8 Rot Angle/deg	17	6	21	4
dHF8Hd-LeuCO/Å	1.77	1.77	1.76	1.76
dHE7H-O1/O2/Å	2.43/1.83	2.31/2.14	2.33/2.17	2.48/2.46

*dX-Y* correspond to distances between X and Y atoms (in Angstroms). *qX* correspond to Mulliken charge of fragment X in ***e*** ^−^ units. HF8 rotational angle is defined respect to an eclipsed conformation. HE7 O1/O2 angles are the ones formed between the Nε of the histidine, the neighbor H atom and the corresponding O atom^26^.

The obtained results show that, as expected, in the R state both subunits display larger ΔE_O2_, and thus slower ligand release rates, which are expected to result in higher affinity. Interestingly, the increase is slightly higher for the β subunits, but both subunits show similar binding energy in both tertiary states. Analysis of the structural parameters also reveal the origin, and subtle differences, in the O_2_ stabilization mechanisms. In the four cases, bound oxygen is forming an H-bond with the imidazole group of distal HE7, mainly as a consequence of the negative charge (qO_2_ < 0) acquired by the ligand upon binding due to the well known π back bonding effect, and reflected in the dHE7H-O1/O2 distances, which range between 1.8 and 2.5 Å. β subunits in both R and T states show larger charge and longer Fe-O distance, a fact that could reflect stronger distal interactions.

Concerning proximal effects, previous work from our group^26^ showed that the key parameter is the Fe-HisF8 distance, which corresponds to 2.12 Å for a geometry optimized isolated heme with the same method. Longer distances indicate that the protein is pushing the histidine away, reducing the O_2_ affinity, while the opposite effect is observed for shorter distances. Subunit αT shows the largest Fe-His distance (2.19 Å), which indicates that in this case structure is pulling HF8 away (possibly reducing oxygen affinity). In the αR state HF8 is allowed to come closer, reaching its optimal distance. In contrast, no difference is observed between the βR and βT structures, the histidine being slightly closer than in the isolated active site in both states. Finally, analysis of HF8 rotational angle and/or HF8Nδ H-bonds, two factors which have also shown to further modulate oxygen affinity, showed no significant differences between different tertiary structures.

#### Proximal HF8 and Distal HE7 contributions

To further evaluate how proximal or distal effects contribute to oxygen affinity in each subunit/state we computed ΔE_O2_ for both HE7G and HF8G + Im mutants (see methods), a strategy which has already proven successful to identify and quantify them^26–28^. In the case of HE7, the mutation simply eliminates the distal H-bond with the bound ligand, while in the case of HF8G + Im, the added free imidazol allows better comparison with wt protein, but destroys protein capacity to control its effect. The results are presented in Table 2.

**Table 2:** Oxygen affinity, selected geometrical parameters and charges for HE7G and and HF8G + Im mutants in α y β subunits, for the T and R states.

	αT	αR	βT	βR
ΔE_wt_/kcal mol^−1^	−23.1	−30.2	−21.7	−30.7
ΔΔE_HE7G_/kcal mol^−1^	−0.1	−0.2	0.9	2.5
ΔΔE_HF8G+Im_/kcal mol^−1^	−4.3	2.8	3.9	4.7
dFe-Im (HF8G)/A	2.12	2.12	2.08	2.09
qO_2_ (HF8G)/e^−^	−0.317	−0.274	−0.328	−0.288
qO_2_ (HE7G)/e^−^	−0.274	−0.222	−0.262	−0.252

Beginning the analysis with the α subunits, it is clear that distal HE7 plays almost no role in stabilizing the bound oxygen. The proximal effect, however, as revealed by the HF8G + Im mutant is striking. While in the αR state, the mutant has a 3 kcal/mol lower affinity, consistent with the close HF8-Fe distance (push effect), in αT state mutant increases the oxygen binding energy in ca. 4 kcal/mol, corroborating that in this case tertiary structure is lowering the affinity by pulling HF8 away. Thus, it is clear that the decrease in the oxygen release rate in the T-to-R transition in the α subunits is direct and exclusively related to the proximal HF8. For the β subunits the picture is more complex since both states show distal and proximal contributions to an increase in affinity, as revealed by the decrease in binding energy in all cases. The decrease is however, slightly larger for the distal effect in the R state (2 vs 1 kcal/mol) partially explaining the overall increase.

#### Dynamical behavior of both Hb quaternary states

To complete the above described tertiary state picture, and analyze dynamics of Hb in both quaternary states (R and T) we performed 100 ns classical MD simulations of both tetramers in the oxygenated as well as their free forms. All four systems are stable during the simulation timescale and remain close to the starting crystal structure (RSMD < 1.8 Å). Analysis of Hb mobility using RMSF plots, shows that α subunits present significantly lower mobility than β subunits, in both R and T states (see figure S1). Distal HE7 hydrogen bond analysis shows that for the T states, in the β subunit the hydrogen bond is slightly more flexible (and possibly weaker), while for the R state no significant differences are observed (see Fig. S2 and Fig. S3).

## Oxygen uptake

### Free energy profiles of oxygen uptake

We now turn our attention to the oxygen uptake process, experimentally characterized by the kinetic association constant (k_on_). As shown in our previous work for Mb, oxygen uptake is tightly connected to the distal HE7 conformation and protonation state. In Mb, HE7 has been shown to be able to adopt both a so-called closed and open conformations, which show a marked difference in the free energy associated to oxygen uptake^24^. To analyze this effect we computed the free energy profile (FEP) of oxygen entry in each subunit/state in both HE7 open and closed conformations.

**Figure 2:**
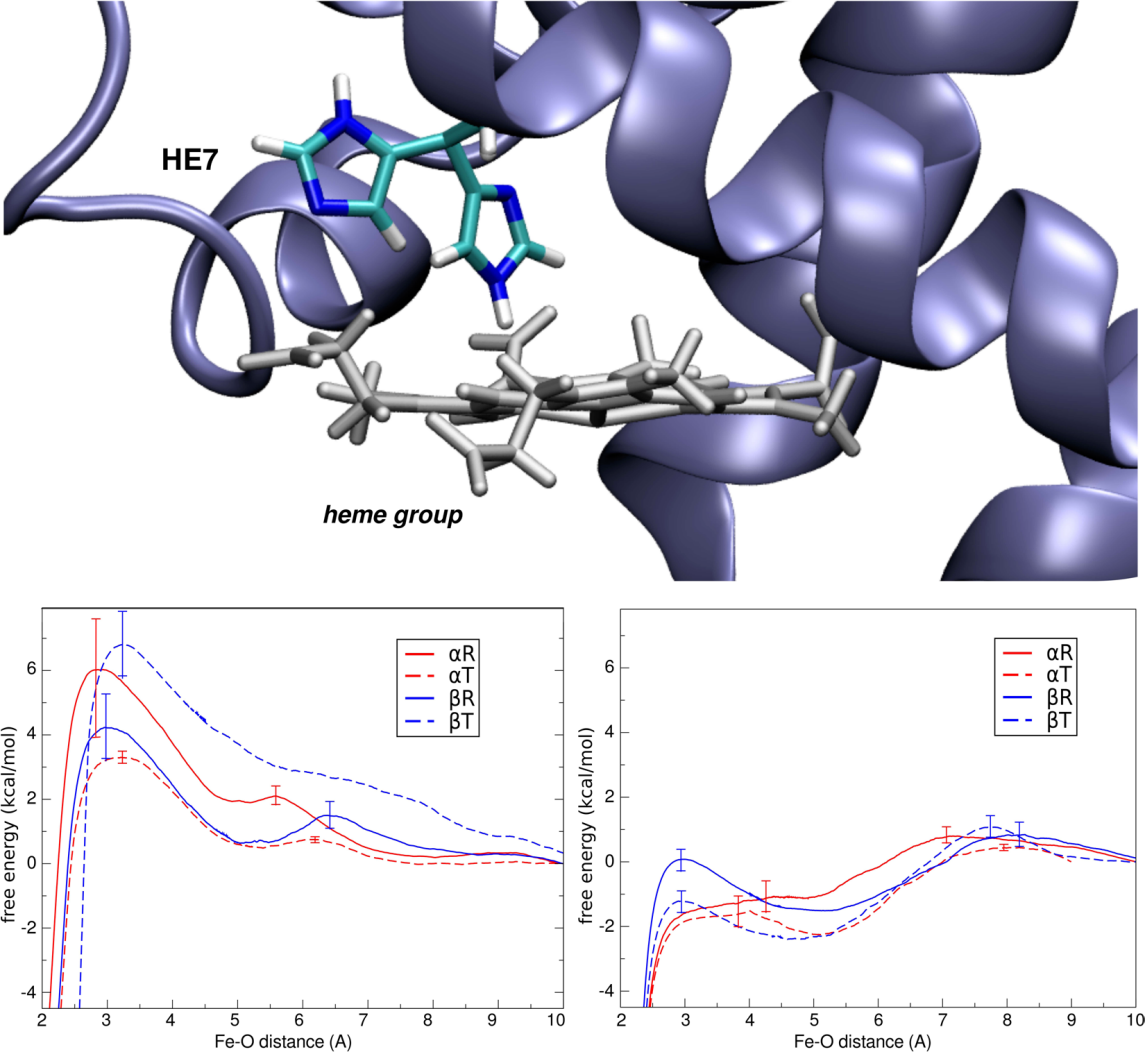
On top, schematic view of the open and closed states of the HE7 gate. On the bottom, free energy profiles for O_2_ migration from the solvent to the distal site in the closed state (left), with HE7 in the HIE protonation state, and in the open state (right), with HE7 in the HID protonation state. For the sake of simplicity, error bars were included only at the Free Energy barriers. In all cases, error estimation resulted lower than 2.1 kcal/mol along the whole free energy profile.

The results presented in Fig. 2 show that, as for Mb, in all four Hb analyzed cases, there is a clear difference between the FEP in the closed and open states, the former displaying barriers in the 4-6 kcal/mol range, while the latter shows very small or no barrier at all. Moreover, also as observed in Mb, HE7 open state results in the creation of a small hydrophobic cavity that draws oxygen inside Hb active site with a a −2 kcal/mol decrease in the free energy. No significant differences are observed between the subunits/states (see S4 and S5 for work distributions and convergence analysis).

### Free energy profiles of HE7 opening

**Figure 3:**
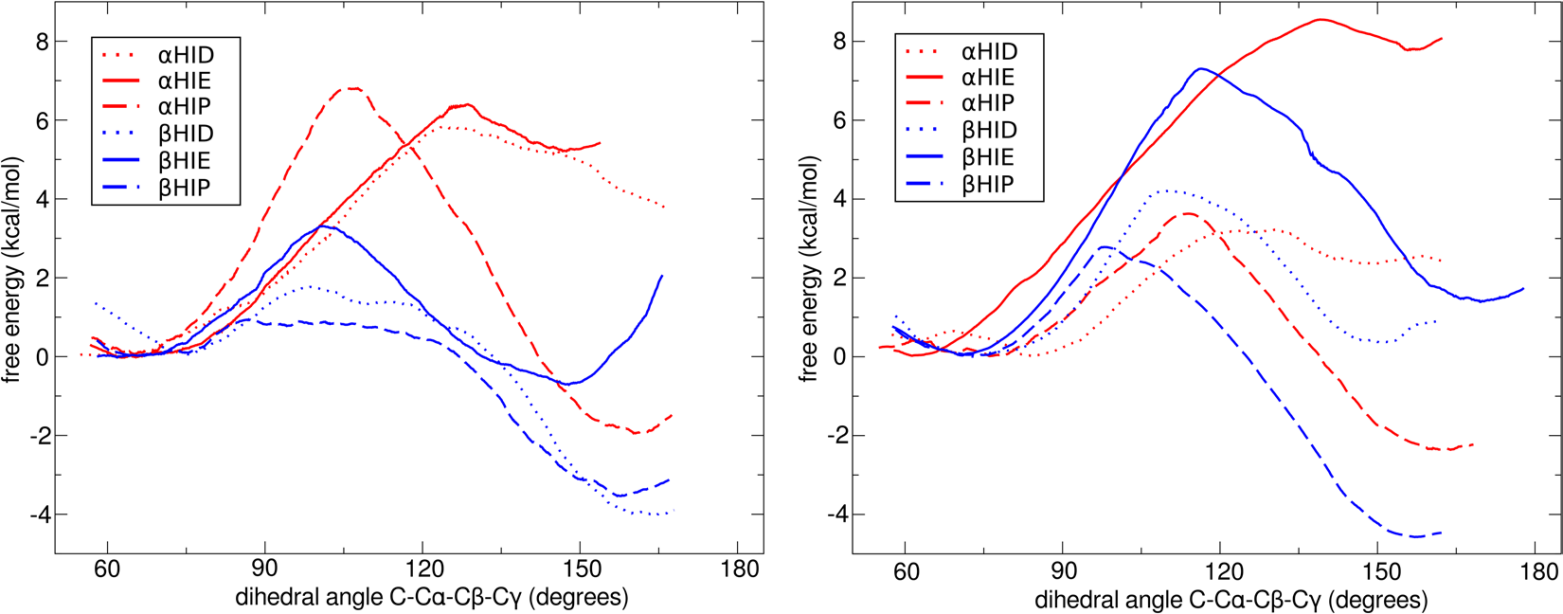
Free energy profiles associated to the HE7 gate opening process in the R (left) and T (right) allosteric states.

Given that, as shown above, oxygen uptake is dependent on HE7 opening, we decided to analyze the free energy associated to this process in each Hb subunit/state, and in all cases considering all three possible histidine tautomers, HID (protonated in δ nitrogen), HIE (protonated in ε nitrogen), and HIP (doubly protonated, with a net positive charge). The resulting FEPs, presented in Fig. 3, show very interesting trends. It is important to make explicit that each free energy profile is independent from the others, and we have translated all closed conformations to the same zero. Those FEP allow us to compare the open and closed conformations, and the barrier between them, and not the relative free energies among all subunits and distal histidine protonation states.

In the T state, both subunits show a similar behavior, with HIE being preferentially closed and with high opening barrier, HID displaying a moderate barrier, and finally the protonated HIP state showing a preference for the open state and smaller barrier. Also important, is that in the T state for all three tautomers β subunits show a favored open state compared to α subunits. In the R state the picture is slightly more complex, α subunits are again predominantly closed in HIE/HID tautomers, and even for the protonated HE7, with a slight preference for the open state, the barrier is quite high. In contrast the β subunits show in all cases small (or completely lack of) opening barrier and the open state is the preferred one in HID and HIP, displaying same free energy in HID.

In summary, while the T-to-R transition does not significantly affect open-close equilibrium in the α subunits, the open fraction -and therefore oxygen uptake- are significantly increased in the β subunits in all considered cases.

## Discussion

**Figure 4:**
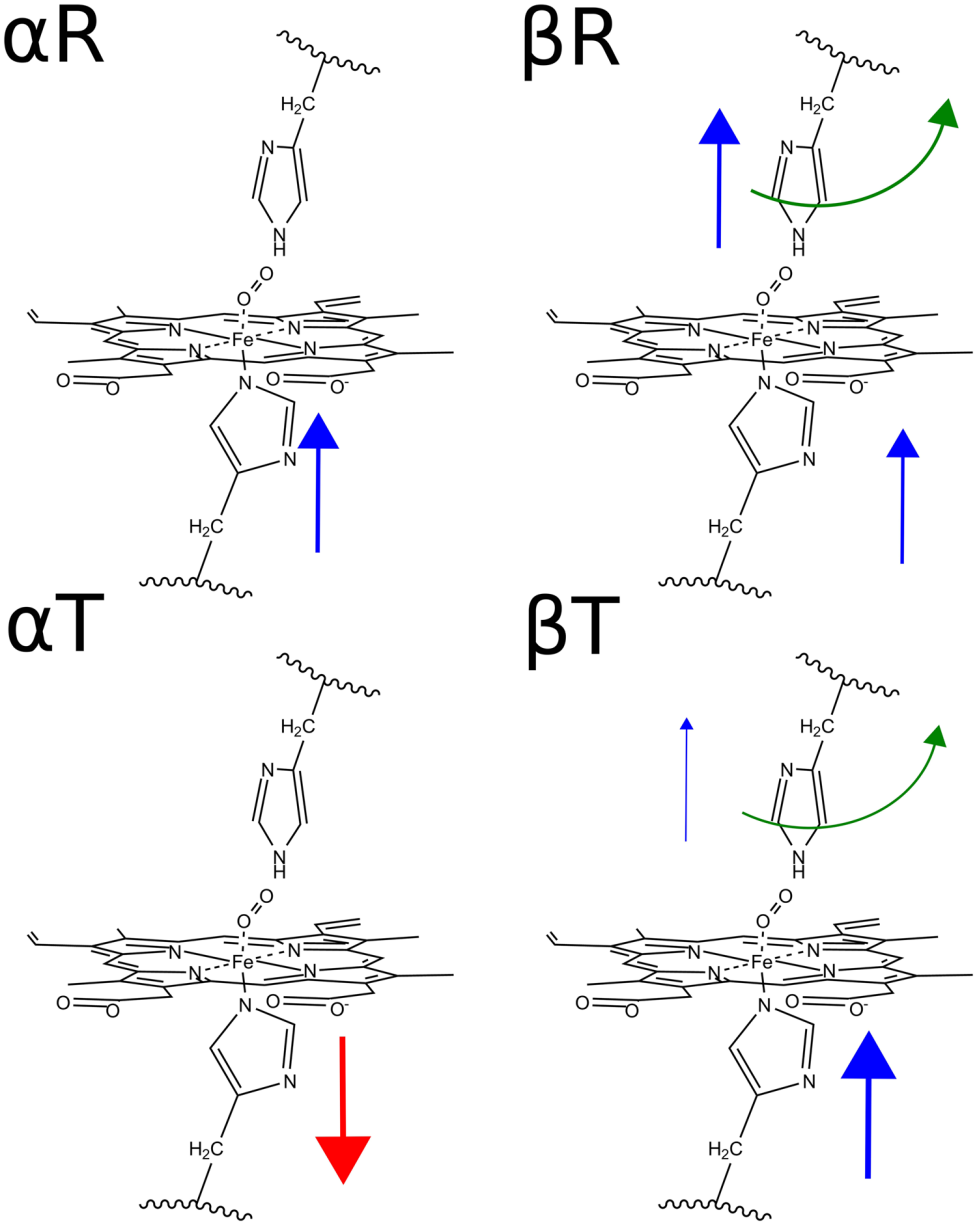
Tertiary mechanisms controlling oxygen affinity in human Hb monomers. Red arrows indicate a negative effect and blue arrows, positive effect. Proximal effects are indicated in the HF8 and distal effects, in the HE7. Curved green arrows in the HE7 show the subunit’s tendency to favor the opened state.

The relevance of human Hb as the paradigmatic, allosteric structurally characterized protein, lead to the performance of the first computational studies of the subject almost 40 years ago by recent Nobel laureates Karplus^54^ and Warshel^55^. Since those times, many Hb simulations were performed, mostly focusing on the allosteric process^30, 56^, paying less attention to how oxygen affinity is differentially regulated. Our results, together with previous works from the group on different members of the globin family, now allow a deeper understanding of the tertiary factors underlying each subunit type and state control of oxygen affinity. A qualitative summary of our results is presented in Fig. 4. Starting from T state, it is evident that in the α subunit low affinity is achieved by a proximal effect that significantly promotes a destabilization of bound oxygen leading to a high k_off_ as experimentally observed. For the β subunit the low affinity results from a complex interplay of proximal, distal and intrinsic effects, which also leads to a high dissociation rate. Also, in the T state HisE7 open conformation is not favored, and the high barrier for oxygen entry in the closed conformation results in slow oxygen entry. The quaternary T-to-R transition, is translated into several effects that altogether increase oxygen affinity. In the α subunit, as expected, the largest and almost exclusive change is related to the proximal HF8 position which now is properly positioned to significantly increase oxygen binding energy. In the β subunit, both proximal and distal effects contribute to the increase in oxygen binding energy consistent with the observed lower k_off_, but also HE7 significantly increases the population of the open state as evidenced by the change in the close-to-open free energy profiles, which results in a net increase in oxygen uptake, again in agreement with the observed increase in k_on_.

Our results can also be analyzed in the context of the early proposal from Perutz^57^ and more recent Resonance Raman studies of hybrid Hbs^58^, computational studies of the allosteric transition^32^, QM/MM studies of CO binding^31^ and the already mentioned TTS model^14^ which together, allowed to establish a connection between Hb reactivity in the context of tertiary and quaternary structural changes. Taken together, those results showed that heme reactivity is controlled exclusively by tertiary forces defining the r and t states, and that quaternary T and R conformations must work by biasing tertiary populations. They also showed that low- and high- affinity tertiary structures, presumably t and r, can coexist within each quaternary state. Our results, show that from an oxygen affinity perspective, crystal structures of each subunit in each quaternary state (αR, αT, βR and βT) are good representatives of the required tertiary low (t) and high (r) affinity structures. Moreover, the changes in affinity are predominantly due to changes in the heme and proximal HF8 -specially in the α subunits- changes that are expected to occur even if quaternary structure is frozen, thus allowing high affinity r-like state to be able to exist in a quaternary T state framework, and vice-versa. Our results, show that from an oxygen affinity perspective, the presently studied crystal structures of each subunit in each quaternary state (αR, αT, βR and βT) are good representatives of the required tertiary low (t) and high (r) affinity structures. In other words, since we observed (and rationalized) known affinity differences between α/βR and α/βT we can reasonably assign them to the “t” and “r” states of the TTS model. And since the changes in affinity are predominantly due to relatively small structural rearrangements in the heme and proximal HF8 (specially in the α subunits). These changes are expected to occur even if quaternary structure is frozen, thus allowing high affinity r-like state to be able to exist in a quaternary T state framework, and vice-versa.

Concerning the observed differences between α and β subunits, early ideas from Perutz, already pointed to proximal effects in the α chains, and distal effects in the β chains, as main contributors to the T-to-R change in affinity. These ideas, received further support from mutagenesis studies of HE7 and VE11^59, 60^, crystal structures of NO bound Hb which highlighted the proximal strain in αT^61, 62^ and above mentioned Raman Studies and QM/MM studies^58^. It is also interesting to note that, in spite of their underlying different mechanisms, both chains show a very similar change in oxygen affinity along the T-to-R transition. This chain equivalence, which was already observed in QM/MM calculations of CO binding^63^ and kinetic measurements^20^, has been suggested as a fundamental requirement for maximum cooperativity. In this context, our data suggest that the smaller proximal effect of the β chains, which allows them to relax -and switch reactivity- faster^58^ is compensated by the distal effect in both k_off_ and k_on_, possibly once full βT to βR transition is achieved.

Also, in relation to the affinity and ligand binding kinetic experiments performed in quaternary trapped structures which supported the TTS mode^18, 64^, our data suggest that most of the change in each subunit oxygen affinity between r and t states, is related to proximal and heme conformation effects, which can be considered small (in terms of atomic displacements) compared to the overall tertiary and quaternary transitions. Therefore, its is not difficult to envisage that a quaternary fixed R state Hb, can allow individual subunits to relax the heme and its proximal His to a t-like state, thus displaying low affinity, as experimentally observed.

Finally, we can also analyze how differential oxygen affinity controlling factors work to achieve positive cooperativity and trigger the R-to-T conformational transition, the hallmarks of Hb function. Starting from deoxy Hb in the T state, first molecular event that takes place when Hb is transferred to a high P_O2_ environment is partial oxygenation. In particular, oxygenation of the α chains most likely results in αT → αR transition due to pull effect of the bound oxygen to proximal HF8 (dFe-NHis distance diminishes from 2.20 to 2.12 Å, and concomitant 0.15 Å Fe in plane displacement). The transition increases oxygen binding energy that results in significant slower release rate. The change now possibly propagates to the β chains, which through the transition, increase their HE7 -gate- opening as revealed by the corresponding free energy profiles, thus promoting oxygen uptake. Finally, oxygen binding to the β chains, relaxes its heme and proximal strains, lowering ligand release and further increasing the affinity. In the reverse scenario, when oxyHb is found in low PO_2_ environment, partially deoxygenation -specially in the α chains-, promotes remaining oxygenated β subunits to change to the t state, decreasing oxygen binding energy and thus promoting its release. In this context, our results not only provide an explanation of how cooperativity is achieved but also point to possible key events that should drive the T-to-R transition.

## Conclusions

Using state of the art MD and QM/MM simulations we have determined the tertiary factors that differentially control subunit (α vs β) and allosteric state (R vs T) dependent oxygen affinity in human hemoglobin, highlighting the relevance of proximal effects in the α chains to drive the transition. Our results further support and strengthen the Tertiary Two State model, by showing the key tertiary factors defining the tertiary “r” and “t” states, and their coexistence in both quaternary states.

## Electronic supplementary material

Supplementary Information
