# Supplementary material for: Tertiary and quaternary structural basis of oxygen affinity in human hemoglobin as revealed by multiscale simulations
Source: Sci Rep. 2017 Sep 7;7:10926. doi: 10.1038/s41598-017-11259-0 (PMC5589765; doi:10.1038/s41598-017-11259-0)
Supplement: Supplementary file 1 — Supplementary Information [file 41598_2017_11259_MOESM1_ESM.pdf]

Tertiary and quaternary structural basis of oxygen affinity in human hemoglobin as revealed by multiscale simulations

Mauro Bringas[12], Ariel A. Petruk[12], Dario A. Estrin[12], Luciana Capece[12]* and Marcelo A. Martí[34]*

Supplementary Information

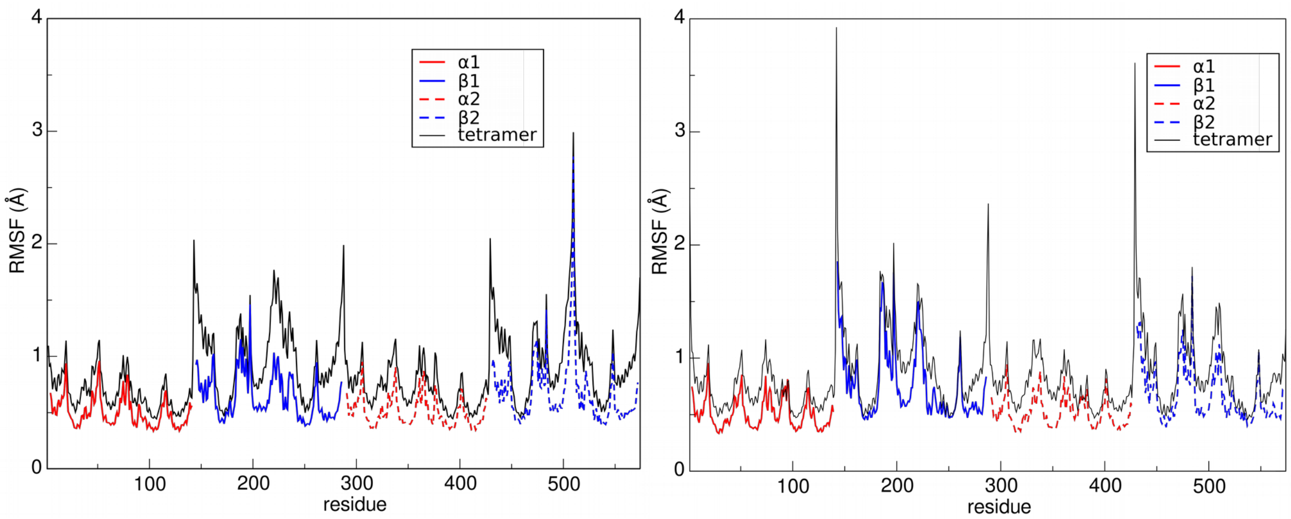

Figure S1. Root of the mean square fluctuations by residue in the R (left) and T (right) states, for each monomer and the whole tetramer.

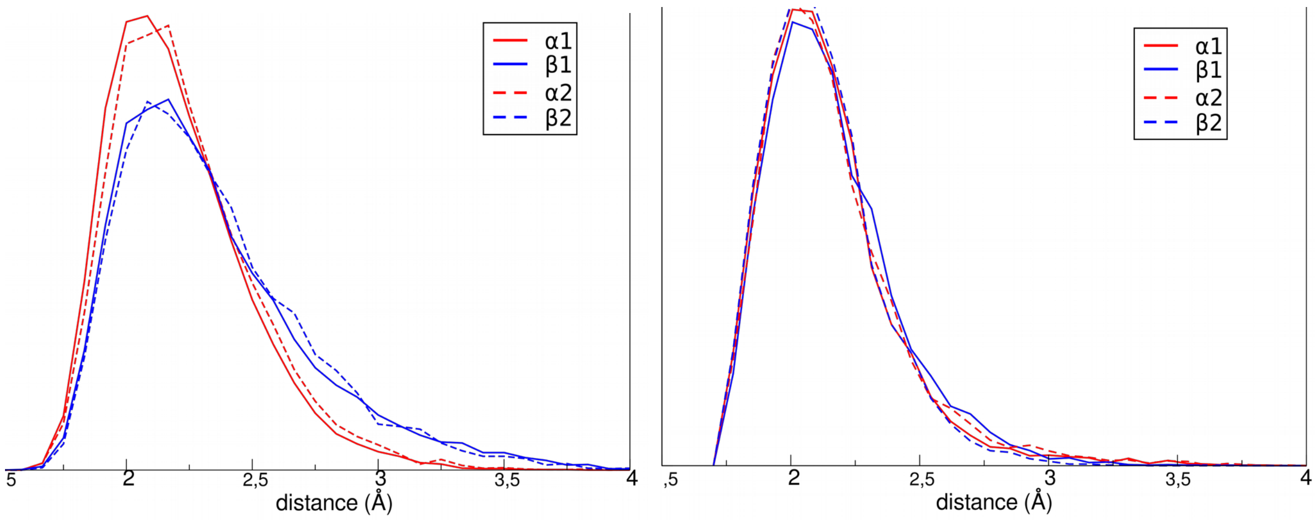

Figure S2. H bond length between HE7 and non coordinated O atom from O2 in the R (left) and T (right) states.

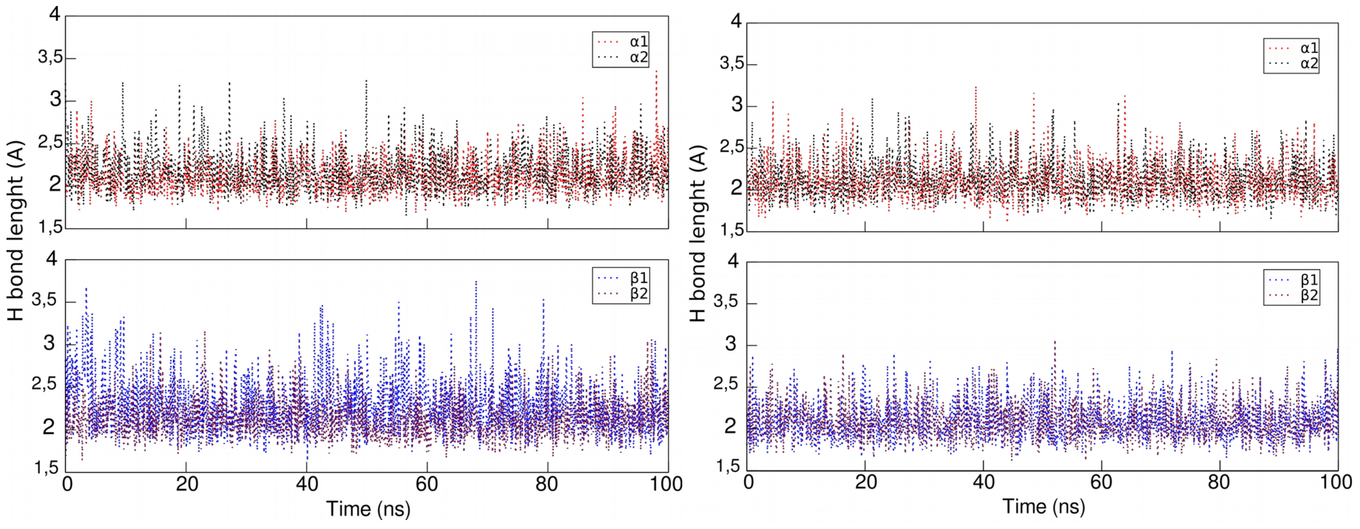

Figure S3. Temporal evolution of H bond length between HE7 and non coordinated O atom from O2 in the R (left) and T (right) states.

Figure S4

a) Non gaussian work distributions for all subunits with the histidine gate closed.

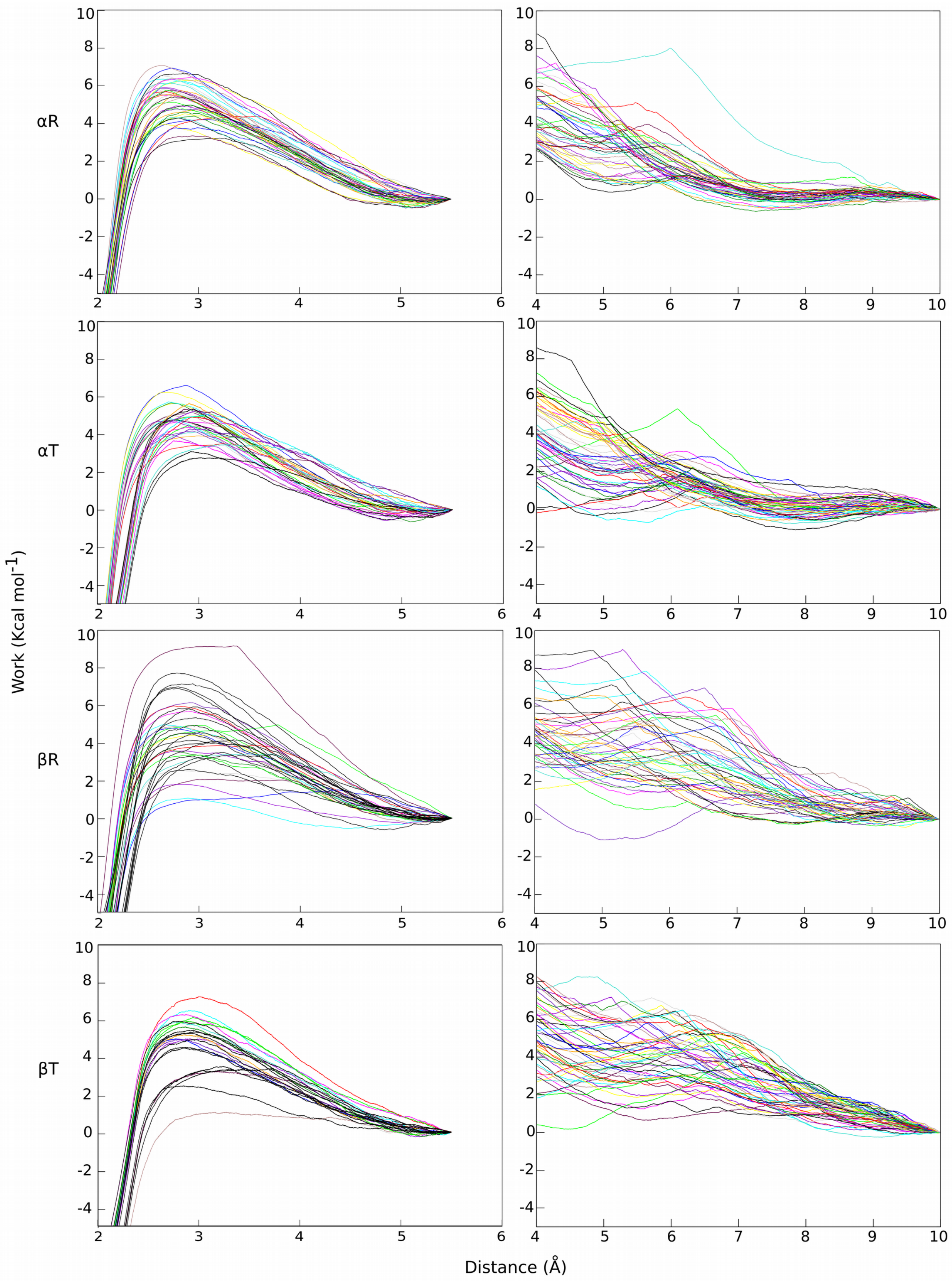

b) Non gaussian work distributions for all subunits with the histidine gate opened.

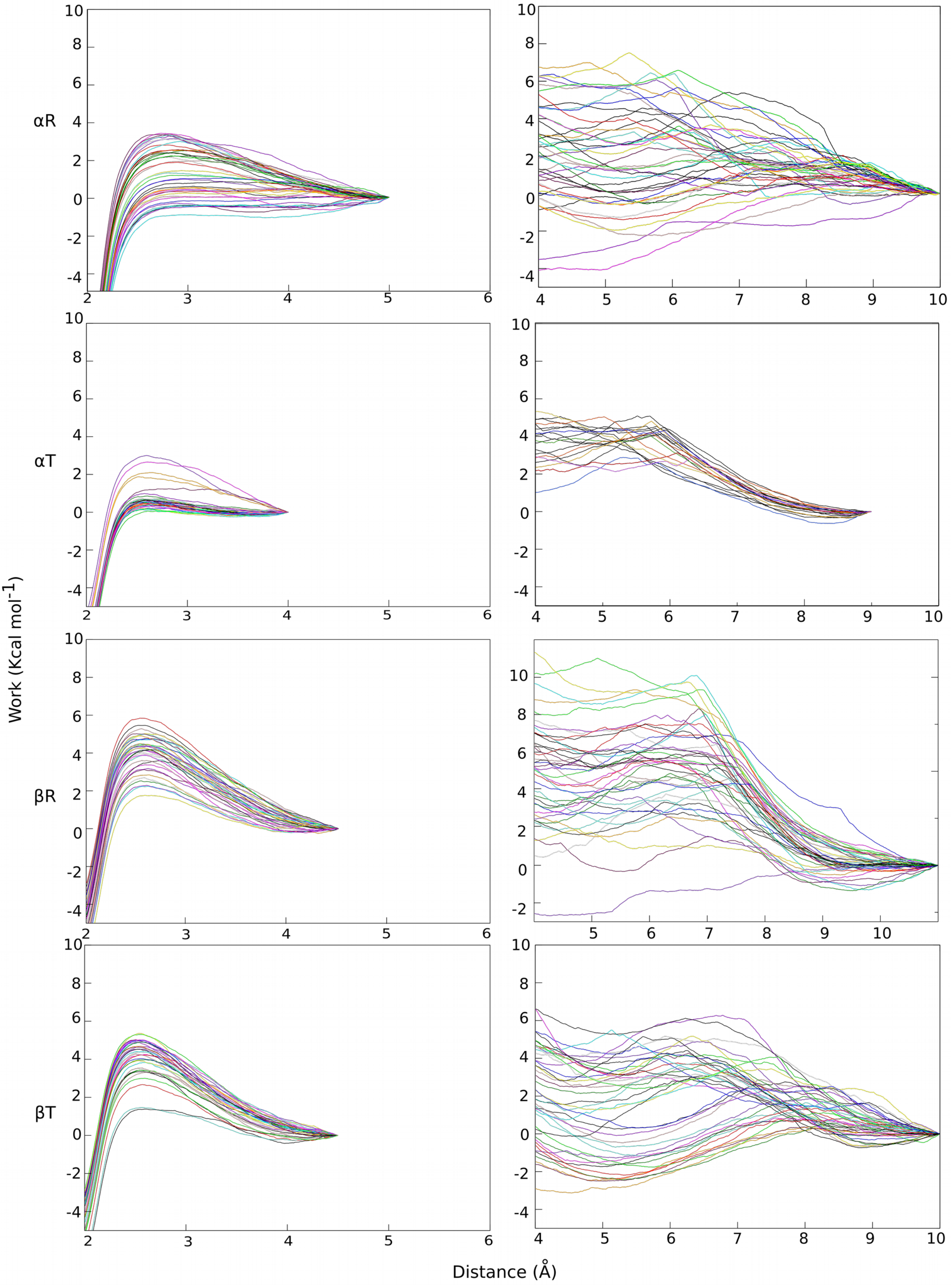

Figure S5

a) Non gaussian Jarzynsky estimator for all subunits with the histidine gate closed considering 50% (red), 75% (blue), 90% (green) and 100% (black) of the work profiles. In all cases, differences between 90% and 100% profiles exponential average do not differ more than 1 kcal/mol.

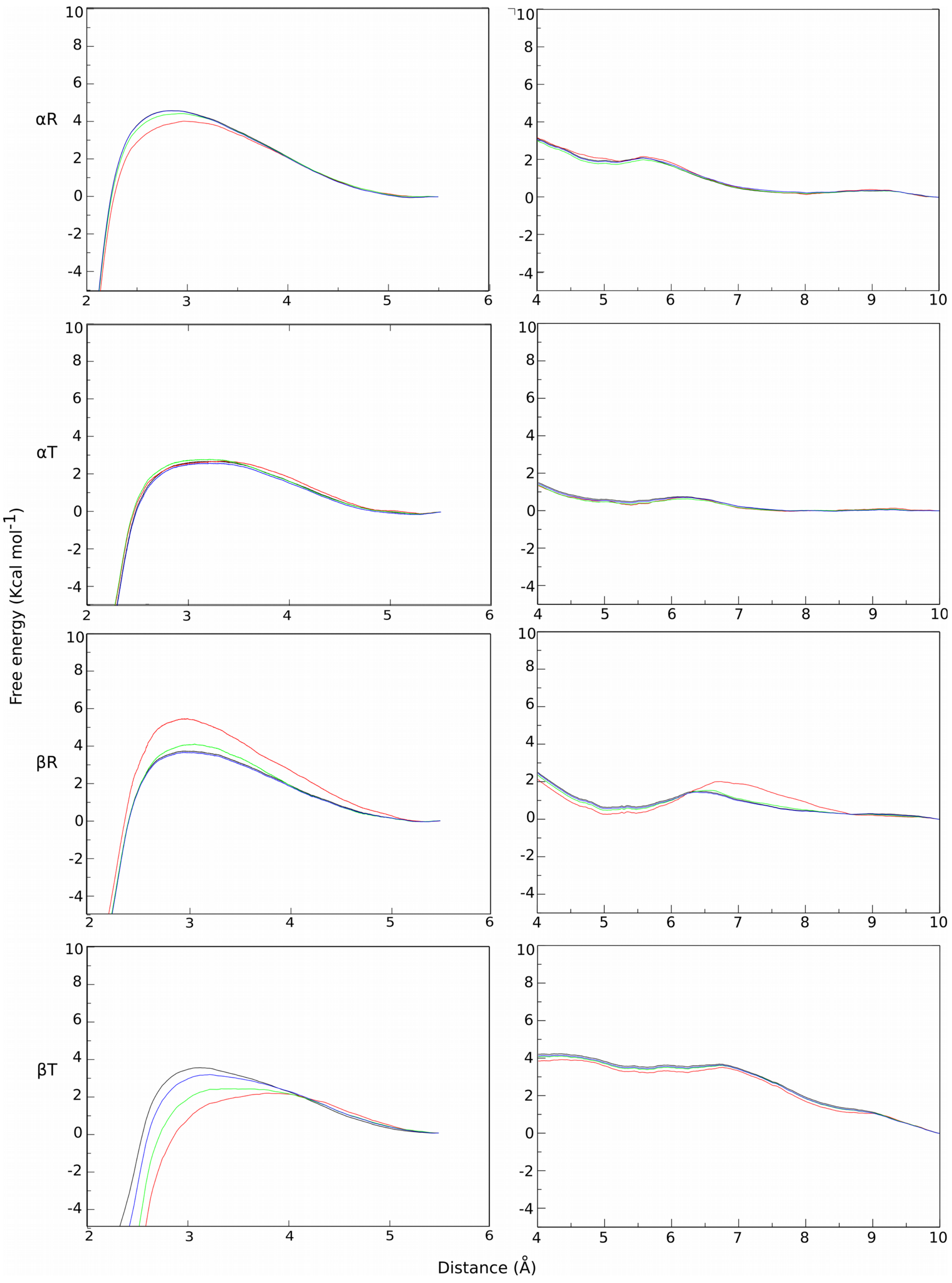

b) Non gaussian Jarzynsky estimator for all subunits with the histidine gate opened considering 50% (red), 75% (blue), 90% (green) and 100% (black) of the work profiles. In all cases, differences between 90% and 100% profiles exponential average do not differ more than 1 kcal/mol.

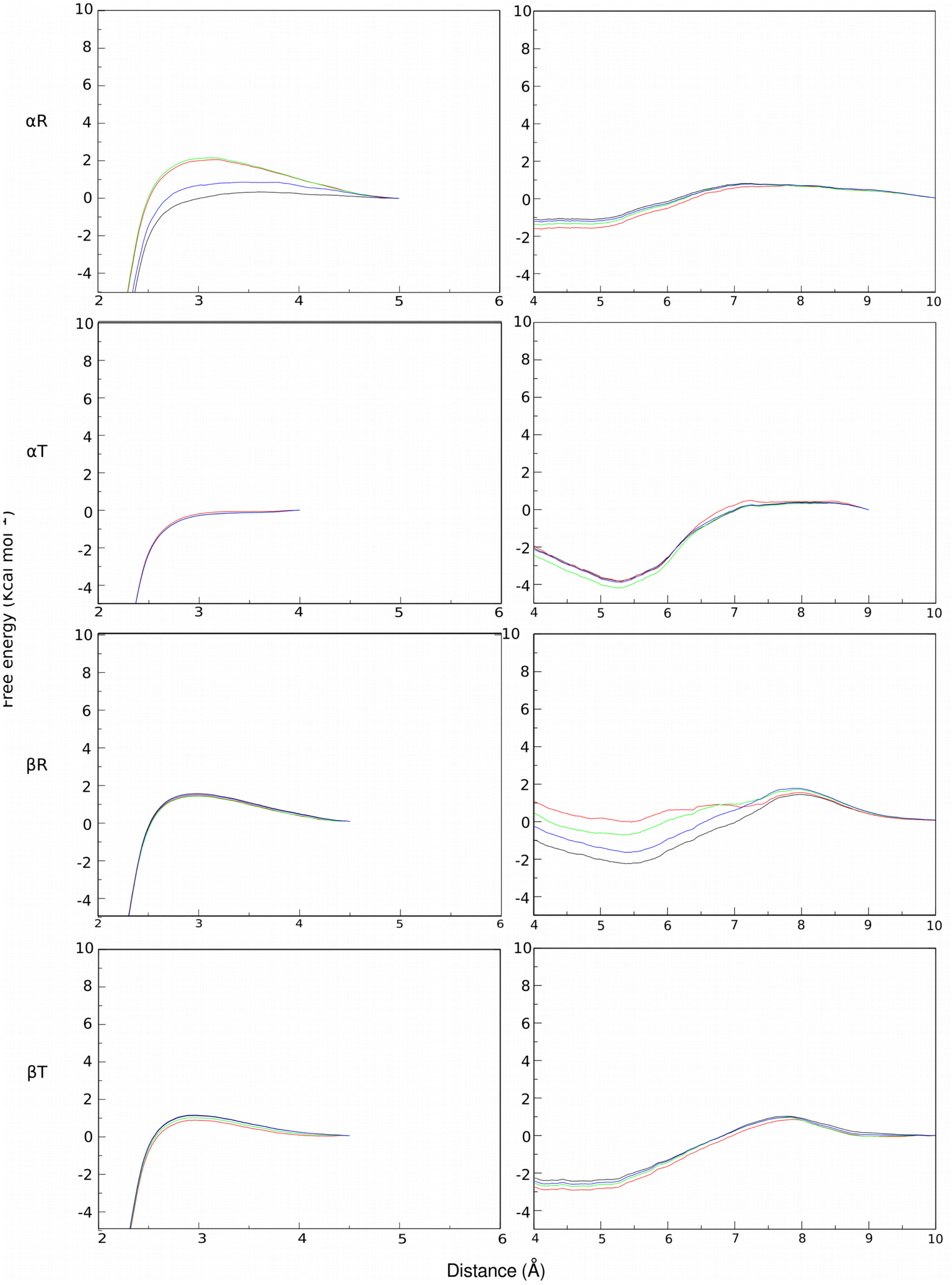

Table S1. Amber-compatible prepi and prm files for deoxy and oxy heme parameters.

```
   0    0    2

Deoxy ferrous heme

HEM  INT     0

CORRECT  OMIT DU   BEG
   0.0000
   1   DUMM  DU    M      0   0   0       0.0000      0.0000      0.0000   0.0
   2   DUMM  DU    M      1   0   0       1.0000      0.0000      0.0000   0.0
   3   DUMM  DU    M      2   1   0       1.0000     90.0000      0.0000   0.0
  19   FE    FE    M     16  14  12       1.0000     90.0000      0.0000   0.3
  20   NA    NP    S     19  16  14       2.0800     98.0000     90.0000   -0.1
  21   C1A   CC    S     20  19  16       1.3800    125.4000     90.0000  -0.045
  22   C2A   CB    B     21  20  19       1.4100    109.0000    180.0000  0.01
  23   CAA   CT    3     22  21  20       1.5100    124.0000    180.0000   0.05
  24   HAA1  HC    E     23  22  21       1.0900    109.5000     60.0000  0.015
  25   HAA2  HC    E     23  22  21       1.0900    109.5000    300.0000  0.015
  26   CBA   CT    3     23  22  21       1.5400    111.0000    180.0000  -0.025
  27   HBA1  HC    E     26  23  22       1.0900    109.5000     60.0000  0.025
  28   HBA2  HC    E     26  23  22       1.0900    109.5000    300.0000  0.025
  29   CGA   C     B     26  23  22       1.5270    109.4000    180.0000  0.635
  30   O1A   O2    E     29  26  23       1.2600    117.2000     90.0000  -0.752
  31   O2A   O2    E     29  26  23       1.2600    117.2000    270.0000  -0.752
  32   C3A   CB    B     22  21  20       1.4100    107.0000      0.0000     0.01
  33   CMA   CT    3     32  22  21       1.5100    125.0000    180.0000  -0.227
  34   HMA1  HC    E     33  32  22       1.0900    109.5000     60.0000   0.07
  35   HMA2  HC    E     33  32  22       1.0900    109.5000    180.0000   0.07
  36   HMA3  HC    E     33  32  22       1.0900    109.5000    300.0000   0.07
  37   C4A   CC    S     32  22  21       1.4100    107.0000      0.0000 -0.045
  38   CHB   CD    B     37  32  22       1.3700    127.0000    180.0000 -0.14
  39   HHB   HC    E     38  37  32       1.0800    120.0000      0.0000 0.15
  40   C1B   CC    B     38  37  32       1.3700    127.0000    180.0000 -0.045
  41   NB    NO    E     40  38  37       1.3800    124.0000      0.0000  -0.1
  42   C2B   CB    B     40  38  37       1.4100    127.0000    180.0000  0.01
  43   CMB   CT    3     42  40  38       1.5100    125.0000      0.0000  -0.17
  44   HMB1  HC    E     43  42  40       1.0900    109.5000     60.0000 0.05
  45   HMB2  HC    E     43  42  40       1.0900    109.5000    180.0000 0.05
  46   HMB3  HC    E     43  42  40       1.0900    109.5000    300.0000 0.05
  47   C3B   CB    B     42  40  38       1.4100    107.0000    180.0000 0.01
  48   CAB   CY    B     47  42  40       1.5100    126.0000    180.0000 -0.08
  49   HAB   HC    E     48  47  42       1.0800    120.0000      0.0000 0.131
  50   CBB   CX    B     48  47  42       1.3300    120.0000    180.0000 -0.38
  51   HBB1  HC    E     50  48  47       1.0800    120.0000      0.0000  0.15
  52   HBB2  HC    E     50  48  47       1.0800    120.0000    180.0000  0.15
  53   C4B   CC    S     47  42  40       1.4100    107.0000      0.0000    -0.045
  54   CHC   CD    B     53  47  42       1.3700    127.0000    180.0000 -0.14
  55   HHC   HC    E     54  53  47       1.0800    120.0000      0.0000  0.15
  56   C1C   CC    B     54  53  47       1.3700    130.0000    180.0000  -0.045
```

```
 57   NC    NP    E     56  54  53       1.3800    124.0000      0.0000  -0.1
 58   C2C   CB    B     56  54  53       1.4100    127.0000    180.0000 0.01
 59   CMC   CT    3     58  56  54       1.5100    125.0000      0.0000  -0.17
 60   HMC1  HC    E     59  58  56       1.0900    109.5000     60.0000  0.05
 61   HMC2  HC    E     59  58  56       1.0900    109.5000    180.0000  0.05
 62   HMC3  HC    E     59  58  56       1.0900    109.5000    300.0000  0.05
 63   C3C   CB    B     58  56  54       1.4100    107.0000    180.0000 0.01
 64   CAC   CY    B     63  58  56       1.5100    126.0000    180.0000 -0.08
 65   HAC   HC    E     64  63  58       1.0800    120.0000      0.0000  0.131
 66   CBC   CX    B     64  63  58       1.3300    120.0000    180.0000  -0.38
 67   HBC1  HC    E     66  64  63       1.0800    120.0000      0.0000  0.15
 68   HBC2  HC    E     66  64  63       1.0800    120.0000    180.0000  0.15
 69   C4C   CC    S     63  58  56       1.4100    107.0000      0.0000  -0.045
 70   CHD   CD    B     69  63  58       1.3700    127.0000    180.0000 -0.14
 71   HHD   HC    E     70  69  63       1.0800    120.0000      0.0000  0.15
 72   C1D   CC    B     70  69  63       1.3700    130.0000    180.0000 -0.045
 73   ND    NO    E     72  70  69       1.3800    124.0000      0.0000  -0.1
 74   C2D   CB    B     72  70  69       1.4100    127.0000    180.0000 0.01
 75   CMD   CT    3     74  72  70       1.5100    125.0000      0.0000 -0.227
 76   HMD1  HC    E     75  74  72       1.0900    109.5000     60.0000  0.07
 77   HMD2  HC    E     75  74  72       1.0900    109.5000    180.0000  0.07
 78   HMD3  HC    E     75  74  72       1.0900    109.5000    300.0000  0.07
 79   C3D   CB    B     74  72  70       1.4100    107.0000    180.0000 0.01
 80   C4D   CC    S     79  74  72       1.4100    107.0000      0.0000 -0.045
 81   CHA   CD    S     80  79  74       1.3700    127.0000    180.0000 -0.14
 82   HHA   HC    E     81  80  79       1.0800    120.0000      0.0000  0.15
 83   CAD   CT    3     79  74  72       1.5100    124.0000    180.0000 0.05
 84   HAD1  HC    E     83  79  74       1.0900    109.5000     60.0000 0.015
 85   HAD2  HC    E     83  79  74       1.0900    109.5000    300.0000 0.015
 86   CBD   CT    3     83  79  74       1.5400    111.0000    180.0000 -0.025
 87   HBD1  HC    E     86  83  79       1.0900    109.5000     60.0000  0.025
 88   HBD2  HC    E     86  83  79       1.0900    109.5000    300.0000  0.025
 89   CGD   C     B     86  83  79       1.5300    109.4000    180.0000  0.635
 90   O1D   O2    E     89  86  83       1.2600    117.2000     90.0000 -0.752
 91   O2D   O2    E     89  86  83       1.2600    117.2000    270.0000  -0.752

LOOP  EXPLICIT
 NA   C4A
 FE   NB
 FE   NC
 FE   ND
 NB   C4B
 NC   C4C
 ND   C4D
 C1A  CHA

IMPROPER
 ND   C1D  C4D  FE
 C1A  C2A  NA   CHA
 C1B  C2B  NB   CHB
 C1C  C2C  NC   CHC
 C1D  C2D  ND   CHD
```

```
 C2A  C3A  C1A  CAA
 C2B  C3B  C1B  CMB
 C2C  C3C  C1C  CMC
 C2D  C3D  C1D  CMD
 C3A  C4A  C2A  CMA
 C3B  C4B  C2B  CAB
 C3C  C4C  C2C  CAC
 C3D  C4D  C2D  CAD
 C4A  NA   C3A  CHB
 C4B  NB   C3B  CHC
 C4C  NC   C3C  CHD
 C4D  ND   C3D  CHA

DONE
STOP
 NA   C1A  C4A  FE
 NB   C1B  C4B  FE
 NC   C1C  C4C  FE
```

```
   0    0    2

  Oxy ferrous heme

heme_all.db3

HEM  INT     0

CORRECT  OMIT DU   BEG

   0.0000

   1   DUMM  DU    M      0   0   0       0.0000      0.0000      0.0000   0.0
   2   DUMM  DU    M      1   0   0       1.0000      0.0000      0.0000   0.0
   3   DUMM  DU    M      2   1   0       1.0000     90.0000      0.0000   0.0
  19   FO    FO    M     16  14  12       1.0000     90.0000      0.0000   0.362
  20   NA    NP    S     19  16  14       2.0800     98.0000     90.0000  -0.1
  21   C1A   CC    S     20  19  16       1.3800    125.4000     90.0000  -0.04
  22   C2A   CB    B     21  20  19       1.4100    109.0000    180.0000  0.02
  23   CAA   CT    3     22  21  20       1.5100    124.0000    180.0000  0.05
  24   HAA1  HC    E     23  22  21       1.0900    109.5000     60.0000  0.015
  25   HAA2  HC    E     23  22  21       1.0900    109.5000    300.0000  0.015
  26   CBA   CT    3     23  22  21       1.5400    111.0000    180.0000  -0.025
  27   HBA1  HC    E     26  23  22       1.0900    109.5000     60.0000  0.025
  28   HBA2  HC    E     26  23  22       1.0900    109.5000    300.0000  0.025
  29   CGA   C     B     26  23  22       1.5270    109.4000    180.0000  0.635
  30   O1A   O2    E     29  26  23       1.2600    117.2000     90.0000  -0.752
  31   O2A   O2    E     29  26  23       1.2600    117.2000    270.0000  -0.752
  32   C3A   CB    B     22  21  20       1.4100    107.0000      0.0000    0.02
  33   CMA   CT    3     32  22  21       1.5100    125.0000    180.0000  -0.227
```

34	HMA1	HC	E	33	32	22	1.0900	109.5000	60.0000	0.07
35	HMA2	HC	E	33	32	22	1.0900	109.5000	180.0000	0.07
36	HMA3	HC	E	33	32	22	1.0900	109.5000	300.0000	0.07
37	C4A	CC	S	32	22	21	1.4100	107.0000	0.0000	-0.04
38	CHB	CD	B	37	32	22	1.3700	127.0000	180.0000	-0.12
39	HHB	HC	E	38	37	32	1.0800	120.0000	0.0000	0.16
40	C1B	CC	B	38	37	32	1.3700	127.0000	180.0000	-0.04
41	NB	NO	E	40	38	37	1.3800	124.0000	0.0000	-0.1
42	C2B	CB	B	40	38	37	1.4100	127.0000	180.0000	0.02
43	CMB	CT	3	42	40	38	1.5100	125.0000	0.0000	-0.17
44	HMB1	HC	E	43	42	40	1.0900	109.5000	60.0000	0.05
45	HMB2	HC	E	43	42	40	1.0900	109.5000	180.0000	0.05
46	HMB3	HC	E	43	42	40	1.0900	109.5000	300.0000	0.05
47	C3B	CB	B	42	40	38	1.4100	107.0000	180.0000	0.02
48	CAB	CY	B	47	42	40	1.5100	126.0000	180.0000	-0.08
49	HAB	HC	E	48	47	42	1.0800	120.0000	0.0000	0.13
50	CBB	CX	B	48	47	42	1.3300	120.0000	180.0000	-0.38
51	HBB1	HC	E	50	48	47	1.0800	120.0000	0.0000	0.15
52	HBB2	HC	E	50	48	47	1.0800	120.0000	180.0000	0.15
53	C4B	CC	S	47	42	40	1.4100	107.0000	0.0000	-0.04
54	CHC	CD	B	53	47	42	1.3700	127.0000	180.0000	-0.12
55	HHC	HC	E	54	53	47	1.0800	120.0000	0.0000	0.16
56	C1C	CC	B	54	53	47	1.3700	130.0000	180.0000	-0.04
57	NC	NP	E	56	54	53	1.3800	124.0000	0.0000	-0.1
58	C2C	CB	B	56	54	53	1.4100	127.0000	180.0000	0.02
59	CMC	CT	3	58	56	54	1.5100	125.0000	0.0000	-0.17
60	HMC1	HC	E	59	58	56	1.0900	109.5000	60.0000	0.05
61	HMC2	HC	E	59	58	56	1.0900	109.5000	180.0000	0.05
62	HMC3	HC	E	59	58	56	1.0900	109.5000	300.0000	0.05
63	C3C	CB	B	58	56	54	1.4100	107.0000	180.0000	0.02
64	CAC	CY	B	63	58	56	1.5100	126.0000	180.0000	-0.08
65	HAC	HC	E	64	63	58	1.0800	120.0000	0.0000	0.13
66	CBC	CX	B	64	63	58	1.3300	120.0000	180.0000	-0.38
67	HBC1	HC	E	66	64	63	1.0800	120.0000	0.0000	0.15
68	HBC2	HC	E	66	64	63	1.0800	120.0000	180.0000	0.15
69	C4C	CC	S	63	58	56	1.4100	107.0000	0.0000	-0.04
70	CHD	CD	B	69	63	58	1.3700	127.0000	180.0000	-0.12
71	HHD	HC	E	70	69	63	1.0800	120.0000	0.0000	0.16
72	C1D	CC	B	70	69	63	1.3700	130.0000	180.0000	-0.04
73	ND	NO	E	72	70	69	1.3800	124.0000	0.0000	-0.1
74	C2D	CB	B	72	70	69	1.4100	127.0000	180.0000	0.02
75	CMD	CT	3	74	72	70	1.5100	125.0000	0.0000	-0.227
76	HMD1	HC	E	75	74	72	1.0900	109.5000	60.0000	0.07
77	HMD2	HC	E	75	74	72	1.0900	109.5000	180.0000	0.07
78	HMD3	HC	E	75	74	72	1.0900	109.5000	300.0000	0.07
79	C3D	CB	B	74	72	70	1.4100	107.0000	180.0000	0.02
80	C4D	CC	S	79	74	72	1.4100	107.0000	0.0000	-0.04
81	CHA	CD	S	80	79	74	1.3700	127.0000	180.0000	-0.12
82	HHA	HC	E	81	80	79	1.0800	120.0000	0.0000	0.16
83	CAD	CT	3	79	74	72	1.5100	124.0000	180.0000	0.05
84	HAD1	HC	E	83	79	74	1.0900	109.5000	60.0000	0.015
85	HAD2	HC	E	83	79	74	1.0900	109.5000	300.0000	0.015

```
 86   CBD   CT    3     83  79  74       1.5400    111.0000    180.0000 -0.025
 87   HBD1  HC    E     86  83  79       1.0900    109.5000     60.0000  0.025
 88   HBD2  HC    E     86  83  79       1.0900    109.5000    300.0000  0.025
 89   CGD   C     B     86  83  79       1.5300    109.4000    180.0000  0.635
 90   O1D   O2    E     89  86  83       1.2600    117.2000     90.0000 -0.752
 91   O2D   O2    E     89  86  83       1.2600    117.2000    270.0000  -0.752
 92   O1    LO    B     19  20  21       1.80      -90.0         0.0   -0.120000
 93   O2    LO    E     92  19  20       1.2       120.0        45.0   -0.180000

LOOP  EXPLICIT
 NA   C4A
 FO   NB
 FO   NC
 FO   ND
 NB   C4B
 NC   C4C
 ND   C4D
 C1A  CHA

IMPROPER
 NA   C1A  C4A  FO
 NB   C1B  C4B  FO
 NC   C1C  C4C  FO
 ND   C1D  C4D  FO
 C1A  C2A  NA   CHA
 C1B  C2B  NB   CHB
 C1C  C2C  NC   CHC
 C1D  C2D  ND   CHD
 C2A  C3A  C1A  CAA
 C2B  C3B  C1B  CMB
 C2C  C3C  C1C  CMC
 C2D  C3D  C1D  CMD
 C3A  C4A  C2A  CMA
 C3B  C4B  C2B  CAB
 C3C  C4C  C2C  CAC
 C3D  C4D  C2D  CAD
 C4A  NA   C3A  CHB
 C4B  NB   C3B  CHC
 C4C  NC   C3C  CHD
 C4D  ND   C3D  CHA

DONE

STOP
```

Amber-compatible prm file for deoxy and oxy heme parameters. Pentacoordinate with histidine in the proximal position or hexacoordinate with histidine in the proximal position and O_2-bound in the distal position.

```
MASS

NP 14.01
NO 14.01
CY 12.0
CX 12.0
CD 12.0
FE 55.0
FO 55.0
LC 12.01
LO 16.00

BOND
FE-NB   60.000     2.17100
FO-NB   60.000     2.07000
FE-NP   50.000     2.09500
FE-NO   50.000     2.09500
FO-NP   50.000     2.02500
FO-NO   50.000     2.02500
FO-LO  200.000     1.74200
LO-LO  848.000     1.27700
FE-LC  200.000     1.75000
LC-LO 1000.000     1.12000
NP-CC  316.000     1.38400
NO-CC  316.000     1.38400
CC-CB  273.000     1.44400
CC-CD  391.000     1.39100
CB-CB  418.000     1.35700
CB-CT  297.000     1.50100
CB-CY  297.000     1.50100
CD-HC  338.000     1.09000
CY-HC  340.000     1.08000
CX-HC  340.000     1.08000
CY-CX  570.000     1.34000
FE-LO    0.000     1.74200

ANGLE

NB-FE-NP    50.000     100.000
NB-FE-NO    50.000     100.000
NB-FO-NP    50.000      87.000
NB-FO-NO    50.000      87.000
NB-FE-NB     0.000     180.000
NB-FE-LC     0.000      90.000
NB-FO-LO     0.000      90.000
NP-FE-NP    50.000     160.000
NO-FE-NO    50.000     160.000
NP-FO-NP    50.000     174.000
```

NO-FO-NO	50.000	174.000
NO-FE-NP	50.000	88.000
NO-FO-NP	50.000	90.000
NO-FE-LC	45.000	90.000
NO-FO-LO	0.000	93.000
NP-FE-LC	45.000	90.000
NP-FO-LO	0.000	93.000
FE-NP-CC	30.000	127.400
FO-NP-CC	30.000	127.400
FE-NO-CC	30.000	127.400
FO-NO-CC	30.000	127.400
NO-CC-CB	70.000	110.300
NP-CC-CB	70.000	110.300
NO-CC-CD	70.000	125.500
NP-CC-CD	70.000	125.500
CC-NP-CC	70.000	105.400
CC-NO-CC	70.000	105.400
CC-CB-CB	70.000	107.000
CC-CD-CC	70.000	124.100
CB-CC-CD	70.000	125.400
CC-CB-CT	70.000	124.900
CB-CB-CT	70.000	128.200
CC-CD-HC	30.000	118.000
CR-NB-FE	0.000	110.000
CR-NB-FO	0.000	110.000
CV-NB-FE	0.000	110.000
CV-NB-FO	0.000	110.000
CB-CT-HC	35.000	109.500
CB-CT-H1	35.000	109.500
CB-CB-CY	70.000	128.200
CB-CY-HC	35.000	120.000
CB-CY-CX	70.000	120.000
CY-CX-HC	35.000	120.000
CC-CB-CY	70.000	124.900
CX-CY-HC	35.000	120.000
CB-CT-CT	63.000	114.000
HC-CX-HC	35.000	120.000
FE-LC-LO	35.000	180.000
FO-LO-LO	0.000	121.000
LP-SH-LP	600.000	160.000
CB-CT-S	50.0	114.70
HC-CT-S	50.0	109.50
CB-CT-S	50.0	114.70
NB-FE-LO	0.0	0.0
NO-FE-LO	0.0	0.0
NP-FE-LO	0.0	0.0
NO-FE-LO	0.0	0.0
NP-FE-LO	0.0	0.0
FE-LO-LO	0.0	0.0

DIHEDRAL

X -NB-FE-X	1	0.000	0.000	2.000

```
X -NB-FO-X    1       0.000       0.000       2.000
X -NO-FE-X    1       0.000     180.000       2.000
X -NO-FO-X    1       0.000     180.000       2.000
X -NP-FE-X    1       0.000     180.000       2.000
X -NP-FO-X    1       0.000     180.000       2.000
X -NO-CC-X    4       5.700     180.000       2.000
X -NP-CC-X    4       5.700     180.000       2.000
X -CC-CB-X    4       3.150     180.000       2.000
X -CB-CB-X    4      21.500     180.000       2.000
X -CD-CC-X    4       7.900     180.000       2.000
X -CB-CT-X    1       0.000     180.000       2.000
X -CB-CY-X    4       0.000     180.000       2.000
X -CY-CX-X    4      30.000     180.000       2.000
X -FO-LO-X    1       0.0       180.0         2.0
X -FE-LC-X    1       0.0       180.0         2.0
X -FE-LO-X    1       0.0         0.0         0.0

IMPROPER
X -X -CC-X    0       1.000     180.000       2.000
X -X -CB-X    0       1.000     180.000       2.000
X -X -NP-X    0       1.000     180.000       2.000
X -X -NO-X    0       1.000     180.000       2.000

NONBON
  FE         1.20000   0.05000   0.00000
  FO         1.20000   0.05000   0.00000
  LO         1.60000   0.20000   0.00000
  LC         1.85      0.12      0.0
  NP         1.8240    0.1700    0.0
  NO         1.8240    0.1700    0.0
  CX         1.9080    0.0860    0.0
  CY         1.9080    0.0860    0.0
```

Amber-compatible prepi and prm files for non ligated O_2.

```
    0    0    2

OXY

OXY   INT     0

CORRECT  OMIT DU   BEG

   0.0000

   1   DUMM  DU    M      0   0   0       0.0000      0.0000      0.0000 0.0
   2   DUMM  DU    M      1   0   0       5.0000      0.0000      0.0000 0.0
   3   DUMM  DU    M      2   1   0       5.0000      90.000      0.0000 0.0
   4   OA    LO    M      3   2   1       5.0000      90.000    180.0000 0.0
   5   OB    LO    E      4   3   2       1.2000      98.0000    90.0000 0.0
```

```
DONE

STOP
```

```
MASS
LO 16.0

BOND
LO-LO   570.0    1.200

ANGLE

DIHEDRAL

IMPROPER

NONBON
LO         1.60000   0.20000   0.00000
```
